# High rate of intestinal parasites among a closed community of Zay populations residing on three islands of Lake Ziway, Ethiopia

**DOI:** 10.1371/journal.pone.0240582

**Published:** 2020-10-22

**Authors:** Haileleul Micho, Mengistu Fantahun, Zenebe Gebreyohannes, Ashenafi Bedaso, Neima Bereka, Bethelehem Abebayehu, Asaye Birhanu Mekonnen, Bineyam Taye, Kassu Desta, Aster Tsegaye

**Affiliations:** 1 Department of Biomedical Sciences, College of Health Sciences, Dilla University, Dilla, Ethiopia; 2 Department of Medical Laboratory Sciences, College of Health Sciences, Addis Ababa University, Addis Ababa, Ethiopia; 3 St. Paul’s Hospital Millennium Medical College, Addis Ababa, Ethiopia; 4 Holy Cross Hospital and Walden University, Minneapolis, Minnesota, United States of America; 5 Colgate University, Hamilton, New York, United States of America; George Washington University School of Medicine and Health Sciences, UNITED STATES

## Abstract

**Background:**

Several factors including socio-economic and access to health facility influence burden of intestinal parasites. Epidemiological data from hard to reach areas will help to identify high-risk communities for targeted intervention. We, therefore, assessed the magnitude of intestinal parasites among Zay people residing in three islands of Lake Ziway in Ethiopia.

**Methods:**

This cross-sectional survey was conducted in March 2013 on 444 individuals aged 6 months to 85 years. Stool samples were analyzed using wet mount and formol-ether concentration methods. Data were collected using interviewer-administered questionnaire and analyzed using STATA version 10.

**Results:**

Among the study participants, 52% (321/444) were children under 15 years. While 72.8% were positive for at least one intestinal parasite, single, dual and triple infections were found in 42.1%, 23.9% and 6.3%, respectively. Four types of intestinal parasites were detected in two children. The commonest parasites were *Entamoeba histolytica/dispar* (51.4%), *Schistosoma mansoni* (17.8%), *Giardia lamblia* (14.4%), *Trichuris trichiura* (10.8%), Taenia species (5.6%), *Hymenolopis nana* (4.5%), *Ascaris lumbricoides* (4.1%), *Entrobius vermicularis* (0.9%), Hookworm (0.7%), and *Strongyloides stercoralis* (0.2%). Remarkable proportion of study participants (51.3%) had no latrine and >85% of the islanders use the lake water for drinking, cleaning or both. About 36% had no information about waterborne and related diseases, while 31% never heard about bilharziasis. Fishing and farming were the main source of income. In the multivariate model, being in the age group > 15 years (AOR = 0.49; 95%CI = 0.28–0.85) and not using lake water for drinking or washing (AOR = 0.52; 95%CI = 0.28–0.99) had protective effect, after adjusting for education, occupation and hand wash after latrine use.

**Conclusion:**

The observed high rate of intestinal parasites (72.8%) in these hard to reach Islanders of Lake Ziway, warrants targeted and sustainable intervention.

## Introduction

Neglected tropical diseases (NTDs) have the greatest relevance for achieving the broader Sustainable Development Goals (SDGs) by 2030 [1]. Among the NTDs, human intestinal parasites still remain as major public health concern. Despite global deworming efforts, as per WHO 2020 estimate, about 1.5 billion people (24% of the world’s population) are infected with soil-transmitted helminthes globally. Infections are widely distributed in the poorest and most deprived communities. The common intestinal parasites are *Ascaris lumbricoides*, *Trichuris trichiura* and hookworms [2]. As stated in a systematic analysis for the Global Burden of Disease Study in 2017 which is based on data from 195 countries globally, Schistosomiasis accounts 143 million, Ascariasis 447 million, Trichuriasis 289 million, and Hookworm disease was reported in 229 million people [3].

Morbidity, mortality and health effects are frequently misconceived and ignored in many developing countries. In sub-Saharan Africa, NTDs are the most common conditions affecting about 500 million people. In total, they produce a burden of disease that may be equivalent to up to one-half of SSA’s malaria disease burden and more than double that caused by tuberculosis. Approximately 85% of the NTD disease burden results from helminthes infections [4, 5]. In tropical zones, intestinal parasitic infections are favored by climatic (temperature and moisture) and socio-economic factors including specific occupations, lack or insufficient sanitation and hygiene, among others [6]. There is a strong association between poverty and prevalence as well as intensity of the infections [7]. Socioeconomic and cultural factors are important for the appearance and spread of intestinal parasites in communities where sanitary conditions and infrastructure are inadequate. Identifying communities where access to clean water, adequate and equitable sanitation and hygiene is limited is an area which needs attention to better understand the magnitude and associated factors. Reaching such hard to reach populations and subgroups by disease programs will be key factor to achieving the 2030 target for the SDGs [1].

Intestinal parasites prevalence rates ranging between 7.4% and 48.2% have been reported from various parts of the developing countries [8–10]. However, prevalence rates as high as 66.3% amongst street children in orphanages in Peru [11], 71.7% in homeless children in Sudan [12] 67.6% in street dwellers in northern Ethiopia [13] have been documented underscoring the need to reach inaccessible communities. A recent study conducted around Lake Zway area revealed prevalence rates of 39.3%, 36.1%, 35.6%, 2.9%, 10.0%, 4.3%, and 2.9%, for *S*. *mansoni*, *Trichuris trichiura*, *Ascaris lumbricoides*, *Enterobius vermicularis*, hookworms, *Hymenolepis nana* and Taenia species, respectively among children [14].

Though in recent years, a decreasing trend in intestinal parasitosis has been documented in Ethiopia, as far as our knowledge goes there is no published study on the magnitude as well as determinants of intestinal parasites including schistosomiasis in areas which are not easily accessible like islands. Thus, this study was carried out among the Zay population residing on three islands of Lake Ziway. Here we hypothesized that intestinal parasite prevalence could be higher among hard to reach communities and use of lake water, lack of access to health facilities and information on hygiene as well as other health risks could be among the determining factors. Understanding the magnitude of common parasitic agents in this isolated population is a first step for developing effective control measures and reducing the associated morbidity. It also helps to minimize the spread of the infection and to develop targeted preventive strategies.

## Methods

### Study setting and context

A cross sectional survey was conducted in March 2013 on the residents of Lake Ziway islands in Ethiopia. Lake Ziway is found in Oromia Region on the border of east Shewa and Arsi zone. It is near to Ziway town, which is located around 160km south of Addis Ababa. There are five islands; named Gelila, Debre Sina, Debretsion (Tullu Gudo), Getie Semani (Funduro), and Debre Abreham (Tedecha), with a population density of around 5, 10, 1000, 105 and 1600, respectively, at the time of the study. Two of the islands, Gelila and Debresina are found near to Ziway town with a distance of approximately 1km from the town. The rest three are relatively far from the town with approximate distance of 18 km (Debretsion), 21km (Getie Semani) and 22km (Debre Abreham). But, they are relatively densely populated and hence selected as study sites. All the Islands are found to the Eastern direction of Ziway town. The people on the islands are called the Zay people having their own unique language Zayigna (Semetic) and are orthodox religion followers. Each island hosts a church or monastery. The total estimate of the Zay population is below 5000. The main source of income for this people is farming and traditional fishing [15, 16].

There were two primary schools on the islands; one on Debre Abreham and the other on Debretsion. Surprisingly, there was no health facility except one health center in Debretsion Island which was almost nonfunctional at the time of this study. As a result, the Zay people were not reached by mass drug administration programs in Ethiopia (after we carried out the study, two health posts, the lowest unit in the Ethiopian health tier system, were established in Debre Abreham (Tedecha) and Debretsion (Tullu Gudo) where Health Extension Workers provide a home to home service). Lack of transportation access left the Zay people isolated from the surrounding population. The traditional boat transport to and from the Ziway town is scheduled once a week. Their interaction with population of the nearby towns Meki and Ziway is increasing from time to time. As there is very limited farming land on the islands and as fish and fish products are being diminished, living on the island is getting very difficult. As a result they are moving or migrating out of the islands for their livelihood. Consequently, their language and culture is at risk of extinction [17]. One of the investigators of this study (H.M) is a native Zay and speaker of Zayigna, who is among the few who had made it to higher education.

### Ethical considerations

The study was conducted after the protocol was ethically reviewed and approved by the Research and Ethical Committee of Department of Medical Laboratory Sciences, College of Health Science, Addis Ababa University (DRERC/17/13/UNDGR, Feb 24/2013). A support letter was written from the department to the local administration to get the necessary support for undertaking the study. Informed written consent was secured from the study participants/parents or guardians for children after providing clear explanation about the purpose and aims of the study. Moreover, assent was obtained from children aged between 12–17 years provided their parents/guardians gave consent. Anti-helminthic drugs were provided for those who were positive for intestinal parasites after consulting clinicians.

### Sample size calculation

The sample size was calculated using single proportion formula based on precision of 5% with 95% confidence level and expected frequency of 50% as follows:

n = (Z_α/2_)^2^ P(1-P)/d^2^

Where;

**n** = minimum sample size;

Ζα/2 = Critical value = 1.96

**p** = proportion of 50%; this value was taken since there were no previous study on the islands

**d** = degree of precision (d = 0.05)

Accordingly, the calculated sample size was 384. Adding a contingency of 10%, the minimum calculated sample size was 422. However, the study recruited 444 participants.

### Sampling technique

The study approached residents of the three islands, namely, Debretsion (Tullu Gudo), Getie Semani (Funduro), and Debre Abreham (Tedecha) based on their closeness to the data collection site conveniently. Stool sample was collected from all of them (n = 444) for whom we also had age and sex data. Whereas for the other variables in the questionnaire, eligible respondents only were responding the questions and the number of respondents for each of them are shown in the result section. Thus, this study employed convenient sampling technique.

### Study population and data collection

The study included residents of the Islands, who stayed in the islands at least 6 months, willing to participate and were capable of providing stool samples. This study excluded those individuals who were moving in and out of the islands for their livelihood in order to get a clear picture about the island residents for designing targeted intervention. Participants were approached through a house to house visit and invited to come to a temporary common site arranged for sample collection considering convenience and ease of access for them. Interviewer administered structured questionnaire was used to collect socio-demographic data and information related to risk factors parents or guardians responded for children. The questionnaire has been translated into the local language “Zayigna” and the commonly spoken language “Amharic” to be used as needed. The questionnaire was prepared by reviewing related studies and pretested in individuals who were not participants of this study. Adjustments were made accordingly to ensure the quality of information gathered.

### Stool specimen collection, preservation and transportation

After obtaining an informed consent and filling the questionnaire, participants were provided with dry, clean, and leak proof plastic stool cup. Instruction on how to collect and bring the specimen was provided. Those who could not produce a stool sample at that point were advised to come the next morning and provide the stool on the spot to avoid delayance. Hence, stool samples were collected from all 444 participants fulfilling the eligibility criteria, labeled with codes and preserved by using SAF (Sodium-acetate-acetic acid formalin). The samples were then transported from the collection site (Three Lake Ziway islands) to Addis Ababa University, School of Medical Laboratory Sciences’ laboratory for analysis using saline wet mount and formol ether concentration techniques.

### Laboratory analysis

#### Wet mount

A size of match stick head stool sample was mixed with a drop of saline on a slide and examined for intestinal parasites under light microscope using a total magnification of 100 x and 400x.

#### Formol ether concentration method

A portion of each preserved stool specimen was taken and processed. Briefly, with an applicator stick a pea sized (approximately 1g) stool sample was emulsified in 4 ml of 10% formol ether water. Then further 3–4 ml of formol ether was added, homogenized and sieved into a clean ether resistant conical centrifuge tube. After adding 3–4 ml of diethyl ether to the formalin solution, the content was mixed and centrifuged at 3200 rpm for 1 minute. The supernatant was poured away with fecal debris. Finally smear was prepared from the sediment and observed under light microscope with a magnification of 100X and 400X to identify ova and larvae.

### Quality assurance

Stool specimens were collected, preserved and transported following standard operating procedures. Samples which were positive by direct and concentration techniques were confirmed by senior medical laboratory technologists and supervisors.

### Statistical analysis

Data were entered using excel sheet, cleaned and analyzed using STATA version 10. Descriptive analysis was employed to determine frequencies and proportions. Association between intestinal parasitosis and independent variables was calculated using chi-square and strength of the association was determined after controlling for confounding factors using multiple logistic regression analysis. P values less than 0.05 were taken as statistically significant.

## Results

### Characteristics of the study participants

A total of 444 individuals residing in three Islands of Lake Ziway and aged 6 months to 85 years were included in this cross-sectional study. The mean (±SD) age was 23.7 years (± 19.3 years) and median 13 years (Inter quartile range IQR = 13–37). As summarized in Table 1, over half of them (52%) belonged to the age group below 15 years, and 50% were males. Majority (49.5%; 194/392) were primary school students while 28.3% (111/392) were illiterate (considering those 5 years and above only). Most of the participants regardless of their occupational status reported fishing as an additional activity while 13.7% (50/366) stated fishing as their primary job. As expected, fish was identified as the main food. About 51.3% (194/378) reported lack of latrine while the majority had a habit of washing their hand after going to toilet (337/379; 88.9%). Shoe wearing was a common habit by the majority of the Islanders (83.5%).

Assessment of lake water usage revealed that, the majority use it for cleaning, drinking or both (85.6%). The study also revealed that the islanders had limited health information as demonstrated by the disclosure of lack of information about waterborne and related diseases by 36% of the participants while 31% never heard about bilharziasis.

**Table 1:** Socio-demographic characteristics of study participants from three islands around Lake Ziway, Oromia Region, Ethiopia, 2013.

Variable	Positive n (%)	Negative n (%)	Total n (%)	P value*
**Sex**				0.337
Male	166 (74.8)	56 (25.2)	222 (50.0)	
Female	157 (70.7)	65 (29.3)	222 (50.0)	
**Age**				0.09
<15	176 (76.2)	55 (23.8)	231(52.0)	
> = 15	147 (69.0)	66 (31.0)	213 (48.0)	
**Education (n = 392)**				0.112
Illiterate	78 (70.3)	33 (29.7)	111(28.3)	
Read and write	39 (72.2)	15(27.8)	54(13.8)	
1–6	155 (79.9)	39 (20.1)	194(49.5)	
7–10	20 (71.4)	8 (28.6)	28(7.1)	
>10^@^	2 (40.0)	3 (60.0)	5(1.3)	
**Occupation (n = 366)**				0.146
Fisher man	35 (70.0)	15 (30.0)	50 (13.7)	
Farmer	41 (71.9)	16 (28.1)	57 (15.5)	
Student	131 (79.4)	34 (20.6)	165 (45.1)	
House wife	63 (67.0)	31 (33.0)	94 (25.7)	
**Latrine (378)**				0.854
Yes	136 (73.9)	48 (26.1)	184(48.7)	
No	145 (74.7)	49 (25.3)	194(51.3)	
**Hand washing after toilet (n = 379)**				0.183
Yes	249 (73.9)	88 (26.1)	337(88.9)	
No	35 (83.3)	7 (16.7)	42(11.1)	
**Shoes wearing (n = 382)**				0.711
Yes	240 (75.2)	79 (24.8)	319(83.5)	
No	46 (73.0)	17 (27.0)	63(16.5)	
**Lake water use (n = 381)**				**0.034**
Yes	251 (77.0)	75 (23.0)	326 (85.6)	
No	35 (63.6)	20 (36.4)	55 (14.4)	

*Chi2 test.

@ 3 diploma; 2 first degree holders.

### Prevalence of Intestinal Parasites (IPs) in the study participants

**Fig 1:**
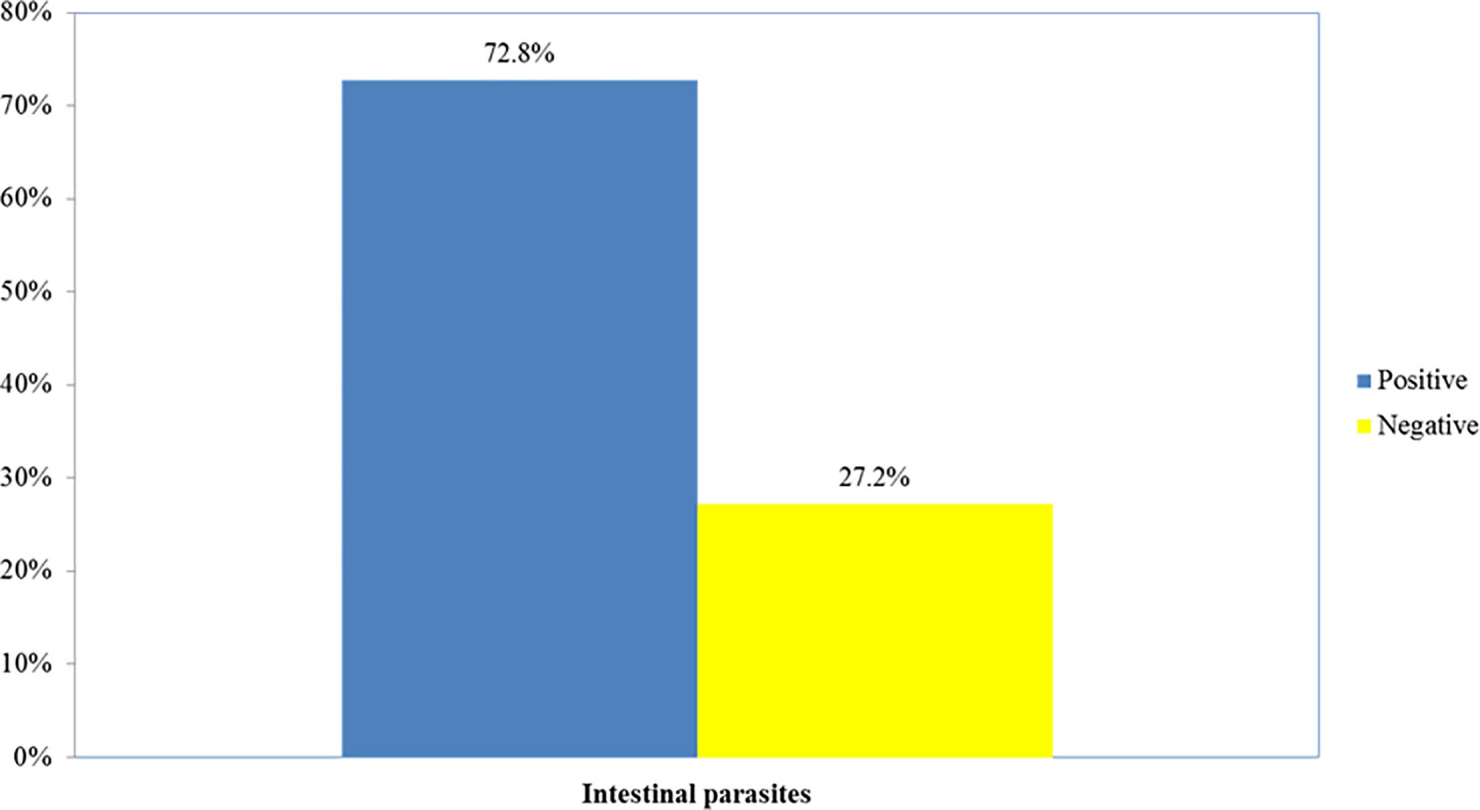
Prevalence of intestinal parasites among populations from three islands of Lake Ziway, Oromia Region, Ethiopia, 2013 (n-444).

**Fig 2:**
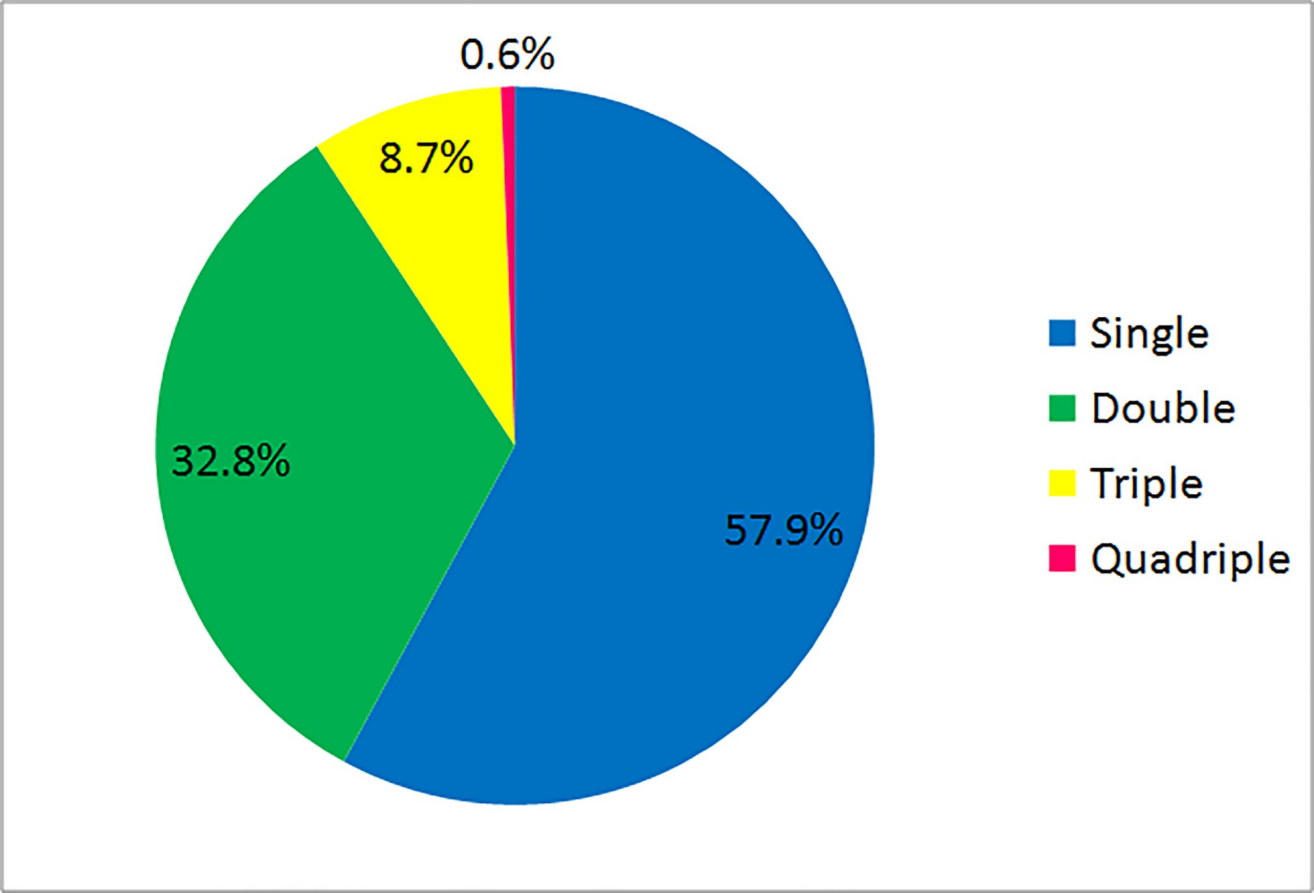
Infection burden of intestinal parasites among populations from three islands of Lake Ziway, Oromia Region, Ethiopia, 2013 (n = 323).

**Fig 3:**
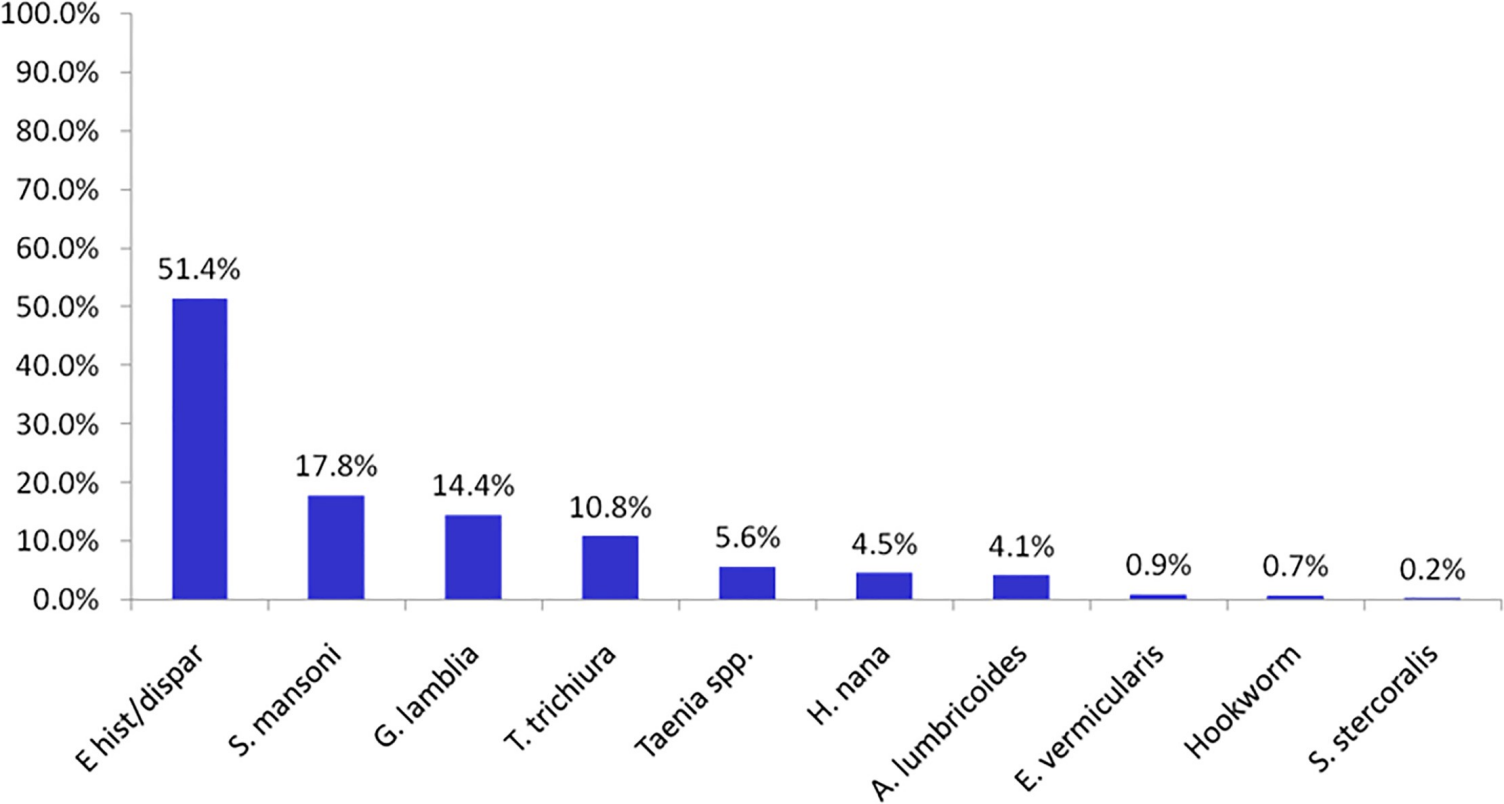
Frequency of intestinal parasites by parasite types among populations from three islands around Lake Ziway, Oromia Region, Ethiopia, 2013 (n = 323).

**Fig 4:**
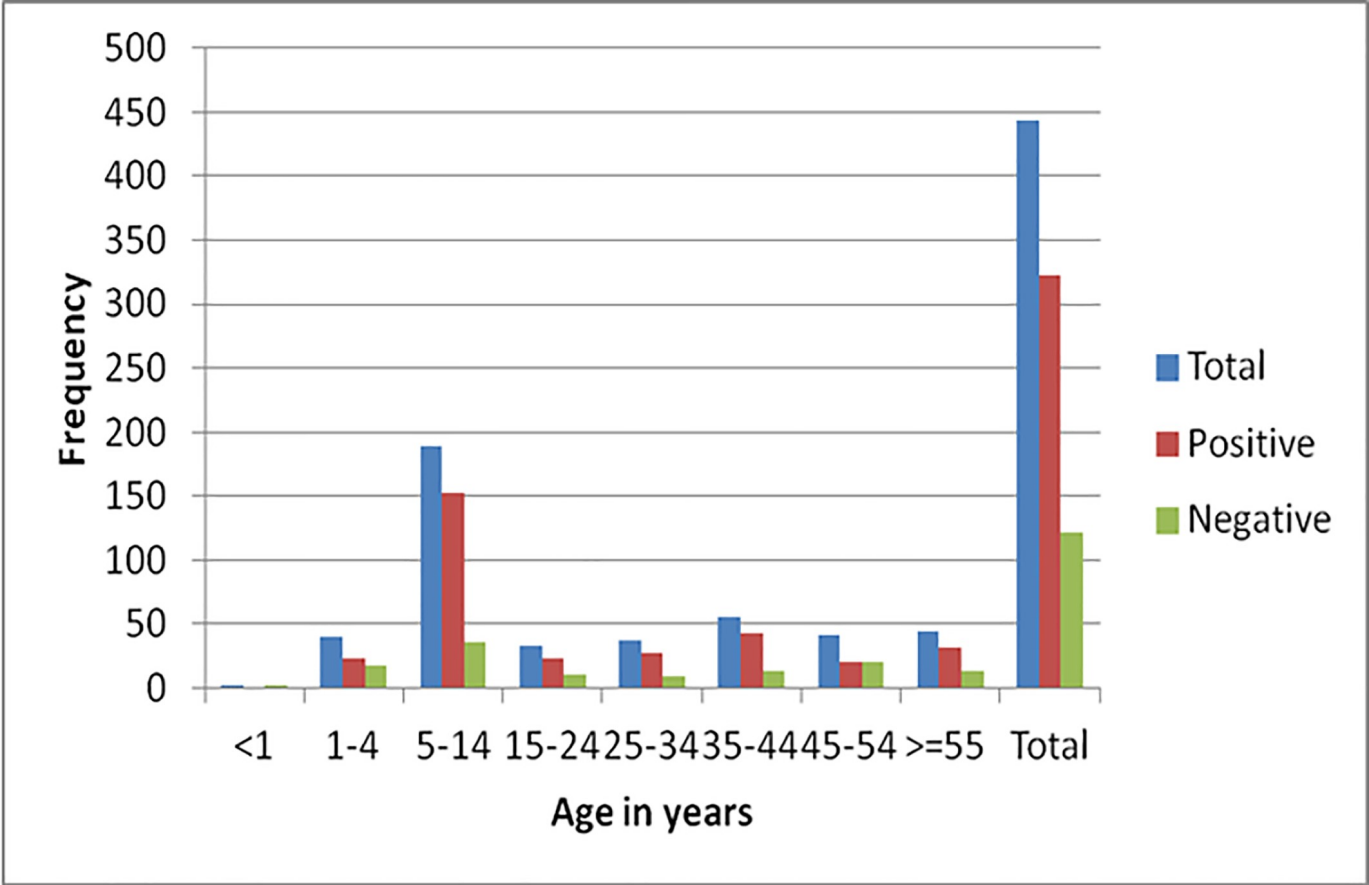
Age specific prevalence of intestinal parasites among populations from three islands of Lake Ziway, Oromia Region, Ethiopia, 2013 (n = 444).

From the total of 444 study participants 72.8% were positive for at least one intestinal parasite (Fig 1). Single, dual and triple infections were found in 57.9%, 32.8% and 8.7%, respectively. Two children aged 7 and 11 years were found harboring 4 intestinal parasites (0.6%) (Fig 2). The most dominant parasitic infections were *Entamoeba histolytica/dispar* (51.4%), *Schistosoma mansoni* (17.8%), *Giardia lamblia* (14.4%), *Trichuris trichuria* (10.8%) (Fig 3). As depicted in Fig 4, age specific analysis revealed a highest frequency of intestinal parasites in the age group 5–15 years (81%, 153/189).

Chi-square analysis revealed no statistically significant association between intestinal parasites positivity and the studied socio-demographic characteristics except for lake water usage (P<0.05) and age which showed marginal significance (P = 0.09), as shown in Table 1.

Analysis of determining factors revealed that none of the selected parameters show a statistically significant association in the multivariate logistic regression model, except being in the age group >15 years [AOR = 0.49; 95%CI = 0.28–0.85] and not using lake water for drinking or washing [AOR = 0.52; 95%CI = 0.28–0.99]. These two variables were found to be protective, after adjusting for education, occupation, hand wash after latrine use, and latrine availability.

## Discussion

Epidemiological study on the prevalence of intestinal parasitic infections in different localities is a primary objective to identify high-risk communities and formulate appropriate intervention. In line with this view, the present study attempted to assess the prevalence of intestinal parasitic infections in Zay people living in three islands of Lake Ziway, the second largest lake in Ethiopia. The study revealed a remarkably high prevalence of intestinal parasites (72.8%), predominated by cyst of *E*. *histolytica/dispar* (51.4%) followed by *S*. *mansoni* (17.8%). The prevalence rate recorded in this study was very high as compared to a study done among Kara and Kwego semi-pastoralist tribes in lower Omo Valley, Southwestern Ethiopia [18]. The difference is more pronounced in the occurrence of *S*. *mansoni* infection (17.8%) which was very high compared to the above mentioned study which identified only one individual parasitized. This may be due to difference in location, awareness of the population, living condition, lack of health service and the high prevalence of vectors (snail) in the island.

As expected for this inaccessible community, the observed rate was much higher compared to reports in other population groups from various parts of developing countries [8–10]. The finding was almost consistent with findings from hard to reach populations like street dwellers [11–13]. Of note, the *S*. *mansoni* infection (17.8%) reported in the current study was lower compared to a recent study around Lake Ziway area which revealed prevalence rates of 39.3%, among the children [14]. The age difference between the two studies could partly explain this difference as the highest prevalence in our study was also noted in the age group 5–14 years. In fact, when we restrict our analysis to school children, the rate of *S*. *mansoni* positivity was 21.2%. This was not unexpected when considering the unavailability of health facilities and the life style of the Zay islanders which is highly dependent on the lake water.

Similar rate of *S*. *mansoni* infection of 21.2% has been detected in school children living around Lake Langano, southeast of Ethiopia. The study from Lake Langano reported a high rate of hookworm (60.2%) [19], unlike the current study in which *E*. *histolytica/dispar* was the highest. Though lower than ours, high rates of 51.7% and 41.5% have also been reported among semi-pastoralist tribes in lower Omo Valley, Southwestern Ethiopia [18].

An earlier cross sectional coproparastiologic study which was conducted among 150 children under the age of 15 who were engaged in fishing and fish processing around Lake Awassa area, south Ethiopia revealed a much higher rate compared to the current study. The overall prevalence for at least one helminthic infection was 92.7%. The most prevalent parasites were *A*. *lumbricoides* (76%), Hookworm species (62.5%), *T*. *trichiura* (60%) and *S*. *mansoni* (33%) [20]. Temporal difference could partly account to this difference as awareness and deworming activities are increasingly practiced from time to time.

Our study tried to determine the associated factors of intestinal parasitic infections among the islanders. Accordingly, being in the age group under 15 years and using lake water for drinking or washing were found to be predictive factors, after adjusting for education, occupation, hand washing habit after latrine use and latrine availability in the multivariate logistic regression model. Young age has been identified as a determinant factor by others as well [21]. It has also been shown that socio-ecological systems govern parasitic infections in humans [22].

On another note, the potential use of parasites as indicators of environmental pollution and the interactions with their hosts has been a topic of interest in the field of environmental parasitology. One review highlights the application of parasites as indicators at different biological scales, from the organismal to the ecosystem [23]. The high rate of intestinal parasites recorded in the Zay islanders of our study, added to their limited access to health care facilities and health information on waterborne diseases could be taken as a potential source of pollutant to Lake Ziway. The Zay people are economically dependent on traditional fishing and farming [16] and use animal dung as their main source of fertilizer [15, 24]; hence they are potentially exposed to parasitic and other infections. Thus, we suggest regular monitoring of the residents and appropriate intervention to protect the lake.

The current study has its own limitation in that it did not quantify the egg count. The study recruited participants conveniently, which may limit generalizability of the finding. As a counter argument, however, only permanent residents who were not moving in and out of the islands for their livelihood and hence are homogeneous in many ways are included. Besides, showing the magnitude and identifying the determining factors for the first time in this hardly accessible population who have limited access to information on waterborne diseases including bilharziasis are part of its strength. The finding will have programmatic impact by drawing attention to such closed communities to ensure the success of the ongoing deworming activities in Ethiopia through expanding and reaching such population groups. It will provide information so that such hard to reach communities are not left out in the global efforts of accelerating progress towards universal health coverage (UHC) and the achievements of the broader Sustainable Development Goals by 2030 [1].

## Conclusion

We observed high rate of intestinal parasitoses (72.8%) in these communities of Lake Ziway Islands in Ethiopia, which are accessible only by using papyrus-made local boats. Young age and use of lake water for drinking and washing were predictive factors. The study also revealed that the Zay people have limited access to health information on waterborne diseases including schistosomiasis. One out of every 5 children was positive for *S mansoni*. The finding warrants targeted intervention. Specifically, expanding the mass deworming activities in the country to such communities, availing functional health facilities, access to health education and clean water for drinking as well as washing must be considered.

## Supporting information

S1 Questionnaire (DOCX)
