# Supplementary material for: High rate of intestinal parasites among a closed community of Zay populations residing on three islands of Lake Ziway, Ethiopia
Source: PLoS One. 2020 Oct 22;15(10):e0240582. doi: 10.1371/journal.pone.0240582 (PMC7580935; doi:10.1371/journal.pone.0240582)
Supplement: S1 Questionnaire — (DOCX) [file pone.0240582.s001.docx]

**S1 Questionnaire.**

**Annex: Questionnaire**

**Addis Ababa University, College of Health Sciences, School of Medical Laboratory Sciences**

Prevalence of intestinal parasite and associated factors among individuals living on islands of Lake Ziway

**1 Socio demographic data**

1.1 sex A. Male B. Female

1.2 Age ­­­­­­­­­­­­­­­­­_______

1.3 Educational status

A, Illiterate B, read and write C, 1-6 D, 7-10 E. Diploma F. Degree

1.4 Occupation A, Fisher man B, Farmer C, Student D, House wife

1.5 Do you have latrine? A, Yes B, No

1.6 If you say yes? A. your own B. Public

1.7 Do you wash your hands after toilet? A, Yes B, No

1.8 Do you wash your hands before eating? A, Yes B, No

1.9 Do you wear shoes? A, Yes B, No

1.10 Do you eat meat? A, Yes B, No

1.11 If you say yes for Q No. 1.10, for how long?

A, daily B, every two days C, every week D, once a month

1.12 Do you use vegetables? A, Yes B, No

1.13 If you say yes for Q No. 1.11, for how long?

A, daily B, every two days C, every week D, once a month

1.14 Do you use the water from the lake? A, Yes B, No

1.15 If yes for what purpose? A, washing B, Drinking C, Both

1.16 Do you have a habit of swimming in the lake? A, Yes B, No

1.17 If yes, how often? A, Daily B, Weekly C, occasionally

1.18 Did you have a habit of eating fish? A, Yes B, NO

1.19 If yes, how often? A, Daily B, Weekly C, occasionally

1.20 What kind of fish? A, Raw B, Cooked C, Both

1.21 Did you have a habit of walking barefoot around the lake? A, Yes B, No

2. **Knowledge assessments**

2.1 Have you ever heard about waterborne and water related diseases? A, Yes B, No

2.2 If you say yes for question no 2.1 from where? A, Radio B, Television C, Community D, News paper E, Others

2.3 Have you /your family ever been sick from waterborne and related disease?

A, Yes B, No C, Do not know

2.4 Symptoms of waterborne and related disease include

A. Abdominal pain B. Diarrhoea C. Weakness D. Don’t know

2.5 Does waterborne and related disease have a treatment?

A. Yes B .No C. Do not know

2.6 Is it possible to prevent waterborne and related disease?

A. Yes B .No C. Do not know

2.7 Which one is/are Preventive mechanisms?

A. Treatment B. Drinking clean water C. Reduce water contact D. Sanitation

E. Killing snails F. Don’t know

2.8 Have you heard about bilharzias (schistosomiasis)? A. yes B. no

2.9 If yes, how do we get bilharzias (schistosomiasis)?

A. Drinking dirty/river water B. Washing in river water C. Contact with contaminated water D. Do not know

1. **ስለ ማህበራዊ ና ግል ሁኔታ መጠይቅ**
   1. **ፆታ 1. ወንድ 2. ሴት**
   2. **ዕድሜ __________**
   3. **የትምህርት ደረጃ 1. ያልተማረ 2. ማንበብና መፀፍ የሚችል 3. 1-6 4.7-10 5. ድፕሎማ 6. ድግሪ**
   4. **ሥራ 1. ዓሣ አጥማጅ 2. ገበሬ 3. ተማሪ 4. የቤት እመቤት**
   5. **ሽንት ቤት አሎት 1. አዎ 2. የለም**
   6. **ካሎት 1. የግል 2. የህዝብ**
   7. **ሽንት ቤት ከተጠቀሙ በኃላ እጆዎትን ይታጠባሉ? 1. አዎ 2. አልታጠብም**
   8. **ከምግብ በፊት እጆትን ይታጠባሉ? 1. እታጠባለዉ 2. አልታጠብም**

**1.9. ጫማ ይጫማሉ (ይለብሣሉ)? 1. እለባሣለዉ 2. አለብስም**

**1.10.ስጋ ይመገባሉ ? 1. እጠቀማለዉ 2. አልጠቀምም**

**1.11. ከተጠቀሙ ለምን ያህል ? 1. በየቀኑ 2. በየሁለት ቀኑ 3. በየሣምንቱ 4. በወር አንዴ**

**1.12. የጓሮ አትክልት ይጠቀማሉ? 1. እጠቀማለዉ 2. አልጠቀምም**

**1.13. ከተጠቀሙ ለምን ያህል ጊዜ? 1. በየቀኑ 2. በየሁለት ቀኑ 3. በየሣምንቱ 4. በወር አንዴ**

**1.14. የሐይቁን ዉሀ ይጠቀማሉ ? 1. እጠቀማለዉ 2.አልጠቀምም**

**1.15. የሐይቁን ዉሃ ለምን ጥቅም ያዉሉታል? 1. ለመታጠብ 2. ለመጠጣት 3. ሁለቱም**

**1.16. ሐይቁን ለዋና ይጠቀሙታል? 1. እጠቀማለዉ 2. አልጠቀምም**

**1.17. አዎ ካሉ 1. በየዕለቱ 2. በየሣምንቱ 3. አልፎ አልፎ**

**1.18. ዓሣ ይመገባሉ ? 1. አዎ 2. አልመገብም**

**1.19. አዎ ካሉ ለምን ያህል ጊዜ ? 1. በየዕለቱ 2. በየሣምንቱ 3. አልፎ አልፎ**

**1.20. ምን አይነት ዓሣ ? 1. ጥሬ 2. የበሰለ 3. ሁለቱንም**

**1.21. ሐይቁ ዳርቻ ላይ በባዶ እግር ይንቀሣቀሣሉ? 1. አዎ 2. አልንቀሣቀሥም**

1. **የዕዉቀት መመዘኛ ጥያቄዎች**

**2.1. ስለ ዉሃ ወላድና ተዛማጅ በሽታዎች ሰምተዉ ያዉቃሉ ? 1. አዉቃለዉ 2.አላዉቅም**

**2.2. በተራ ቁጥር 2.1 መልሶት አዎ ከሆነ ከየት ሰሙ ከአንድ መልስ በላይ መምረጥ ይቻላል?**

**1. ሬድዮ 2. ቴሌቪዥን 3. ከህዝብ 4. ጋዜጣ 5. ሌሎች**

**2.3. እርሶ ወይም የእርሶ ቤተሰብ በዉሃ ወለድና ተዛማጅ በሽታዎች ታመዉ ያዉቃሉ?**

**1. አዎ 2. አይደለም 3. አላዉቅም**

**2.4. የዉሃ ወለድና ተዛማች በሽታዎች ምልክት?**

**1. የሆድ ህመም 2. ተቅማጥ 3. ድካም 4. አላዉቅም**

**2.5. የዉሃ ወለድና ተዛማች በሽታዎች መድሃኒት አላቸዉ?**

**1. አዎ 2. አይደለም 3. አላዉቅም**

**2.6. የዉሃ ወለድና ተዛማች በሽታዎችን መከላከል ይቻላል?**

**1. አዎ 2. አይደለም 3. አላዉቅም**

**2.7. የመከላከያ ዘዴ የሆኑት የትኞቹ ናቸዉ?**

**1. ህክምና 2. ንፁህ ዉሃ መጠጣት 3. ከዉሃ ጋር ያለንን ግንኙነት መቀነስ 4 . ንፅህናንመጠበቅ 5. ቀንዳዉጣ መግደ ል 6. አላዉቅም**

**2.8. ስለ ብላርዝያ ሰምተዉ ያዉቃሉ? 1. አዉቃለሁ 2. አላዉቅም**

**2.9**. **ብላርዝያ እንዴት ልይዘን ይችላል?**

**1. ንፁህ ያልሆነ ዉሃ በመጠጣት 2. የወንዝ ዉሃን በመጠቀም 3 . ከተበከለ ዉሃ ጋር ንክኪ 4. አላዉቅም**
